# Molecular genetics of human primary microcephaly: an overview

**DOI:** 10.1186/1755-8794-8-S1-S4

**Published:** 2015-01-15

**Authors:** Muhammad Faheem, Muhammad Imran Naseer, Mahmood Rasool, Adeel G Chaudhary, Taha A Kumosani, Asad Muhammad Ilyas, Peter Natesan Pushparaj, Farid Ahmed, Hussain A Algahtani, Mohammad H Al-Qahtani, Hasan Saleh Jamal

**Affiliations:** 1 Department of Biochemistry, Faculty of Science, King Abdulaziz University, KSA; 2 Center of Excellence in Genomic Medicine Research, King Abdulaziz University, KSA; 3 KACST Technology Innovation Center in Personalized Medicine, King Abdulaziz University, Jeddah, Kingdom of Saudi Arabia; 4 King Fahd Medical Research Center, King Abdulaziz University, KSA; 5 King Saud bin Abdulaziz University for Health Sciences, KSA; 6 Department of Obstetrics and Gynecology, King Abdulaziz University Hospital, Jeddah, Kingdom of Saudi Arabia

## Abstract

Autosomal recessive primary microcephaly (MCPH) is a neurodevelopmental disorder that is characterised by microcephaly present at birth and non-progressive mental retardation. Microcephaly is the outcome of a smaller but architecturally normal brain; the cerebral cortex exhibits a significant decrease in size. MCPH is a neurogenic mitotic disorder, though affected patients demonstrate normal neuronal migration, neuronal apoptosis and neural function. Twelve MCPH loci (MCPH1-MCPH12) have been mapped to date from various populations around the world and contain the following genes: *Microcephalin*, *WDR62*, *CDK5RAP2*, *CASC5*, *ASPM*, *CENPJ*, *STIL*, *CEP135*, *CEP152*, *ZNF335*, *PHC1* and *CDK6*. It is predicted that MCPH gene mutations may lead to the disease phenotype due to a disturbed mitotic spindle orientation, premature chromosomal condensation, signalling response as a result of damaged DNA, microtubule dynamics, transcriptional control or a few other hidden centrosomal mechanisms that can regulate the number of neurons produced by neuronal precursor cells. Additional findings have further elucidated the microcephaly aetiology and pathophysiology, which has informed the clinical management of families suffering from MCPH. The provision of molecular diagnosis and genetic counselling may help to decrease the frequency of this disorder.

## Introduction

In this review article, we discuss the clinical manifestations of autosomal recessive primary microcephaly (MCPH), its incidence, and molecular genetics, including a comprehensive review of the twelve known mapped loci (MCPH1-MCPH12). Further, we review the corresponding genes and the proteins encoded by these genes, their possible role in the developing brain and reported mutations of these genes. In addition, the potential for these genes to perform various cognitive roles during human brain evolutionary processes is discussed.

## Clinical features of MCPH

Microcephaly is characterised by a reduced occipitofrontal circumference (OFC) of the head that is at least 4 standard deviations (SD) and is caused by congenital insufficiency during fetal brain development, which chiefly affects the cerebral cortex. The current clinical definition of MCPH is as follows: A: a head circumference (HC) of at least 4 SD below the age- and sex-matched means [1]; B: intellectual impairment that is not related to a neurological finding, such as spasticity or progressive cognitive decline [1]; and C: most MCPH patients are of a normal height, weight, and appearance and have normal chromosome analysis and brain scan results [2,3]. The only exception of this is observed in patients with mutations in the microcephalin gene who have a clinically defined short stature and abnormal chromosome analysis results [4-6]. Microcephaly in MCPH is primary, is evident by the 32nd week of gestation, can be observed at the time of birth and is non-progressive [7,8]. HC or OFC is the most common diagnostic tool used for MCPH. Based on the findings of several studies conducted on more than 200 families worldwide, it is believed that the specific physical appearance of patients linked to each MCPH locus is indistinguishable [9-12]. Additionally, the chromosomal analysis for most MCPH patients was reportedly normal [9]. However, recent studies supported by modern neurodiagnostic modalities, such as computed tomography (CT) scan and magnetic resonance imaging (MRI), have significantly increased our knowledge regarding the discernible clinical presentations of MCPH and have expanded the definition of MCPH based on phenotype [7,13,14].

## Molecular Genetics of MCPH

### MCPH Incidence

Primary microcephaly is present at birth and is a static developmental anomaly, whereas secondary microcephaly develops postnatally and is a progressive neurodegenerative condition. Primary microcephaly is very rare, and the rate of incidence is estimated to be 1 in 30,000 in Japan [15] and 1 in 250,000 in Holland [16]. The incidence is increased up to 1 in 10,000 in regions where marriages between cousins are common [1,6]. The birth incidence of primary microcephaly differs from 1.3 to 150/100,000, depending on the population type and consanguineous populations [17].

### MCPH Inheritance

MCPH is inherited in an autosomal recessive pattern in which both copies of the gene in each cell have mutations. Individuals with MCPH inherit one copy of the mutated gene from each parent, but the parents typically do not demonstrate any signs or symptoms of the condition [18].

### Genes in MCPH

MCPH is a neurogenic mitotic disorder that does not occur due to abnormal neuronal migration or neural apoptosis. Rather, it develops because all of the identified MCPH genes are expressed in the neuroepithelium; the phenotypic features and brain scans of MCPH patients reveal a smaller than average brain size. Brain size during birth is determined by both the proliferation rate and cell death rate during neurogenesis. The majority of neurons originate from neural progenitors, which are the predominant cells of the neuroepithelium (NE) lining the brain ventricles [1]. The precise mitotic activity of neuronal progenitor cells was first described in fly neuroblasts: *symmetrical* cell divisions with mitotic spindles oriented in the neuroepithelium plane produce two neuronal progenitor cells, whereas *asymmetric* cell division occurs when the mitotic spindles are perpendicularly oriented toward the neuroepithelium and results in one postmitotic neuron and one progenitor cell [1]. Because the timing of these divisions during development is critical for the final number of neurons, any change in its regulation could lead to cortical disorders, such as microcephaly [19]. MCPH exhibits genetic heterogeneity, and to date, twelve loci (MCPH1-MCPH12) have been associated with this condition [20]. A comprehensive review is outlined below of the twelve MCPH genes that carry mutations, their possible functions in causing the disease (Table 1), various mutations in people of different ethnic backgrounds (Table 2) and animal model studies (Table 3) of the MCPH genes.

**Table 1:** Summary of known MCPH genes, their location and role.

Locus	Gene	Cytoband	Cellular location	Role/effect on brain development	References
MCPH1	Microcephalin	8p23.1	Nucleus	Involved in chromosomal condensation, reduced MCPH1 enhances the production of early born neurons, which comprise deep layers (IV–VI), and reduces the late-born neurons, which produce the thinner outer cortex layer (II–III).	[6,27]
MCPH2	*WDR62*	19q13.12	Nucleus/Centrosomes/Neuronal precursors/Post-mitotic neurons	Cerebral cortical development, proliferation and migration of neuronal precursors, mutation in *WDR62* affects its role in proliferating and migrating neural precursors and causes severe brain malformations.	[13,30]
MCPH3	*CDK5RAP2*	9q33.2	Centrosome	Regulates microtubule function, mutation in *CDK5RAP2* reduces the progenitor pool/decreases the number of neurons and reduces cell survival.	[35,36,38,88]
MCPH4	*CASC5*	15q15.1	Kinetochore	Vital for the spindle checkpoint of the mitotic cycle, *CASC5* underscores the role of kinetochore integrity in the proper volumetric development of the human brain.	[40,41]
MCPH5	*ASPM*	1q31.3	Pericentrosomal	Orientation of mitotic spindles during embryonic neurogenesis, *ASPM* mutations can decrease the size of the brain by influencing the orientation of the mitotic spindle.	[57,54,14]
MCPH6	*CENPJ*	13q12.12	Centrosome/neuroepithelium of the frontal cortex	Controls centriole length/microtubule function, its deletion causes an increased incidence of multiple spindle poles, apoptosis and mitosis arrest	[63,64]
MCPH7	*STIL*	1p33	Pericentrosomal	Apoptosis regulator/cell cycle progression, its mutation in zebrafish causes an embryonic lethal defect, and *STIL* knockout mice (*Sil-/-*) exhibit numerous developmental abnormalities/decreased size/defective midline neural tube.	[72,69,68]
MCPH8	*CEP135*	4q12	Centrosome	Maintains organisation/structure of the centrosome, *CEP135* knockdown showed decreased growth rate/disorganised microtubules.	[72,70,20]
MCPH9	*CEP152*	15q21.1	Centrosome	Centriole duplication/shape to cell/polarity/motility, conversion of glutamine into proline disturbs potential coiled-coiled protein domain/reduced head size.	[76,77]
MCPH10	*ZNF335*	20q13.12	Nucleus	Progenitor cell division/differentiation, mutated *ZNF335* gene causes degeneration of neurons, knockdown of *ZNF335* caused a small brain size with an absent cortex/disrupted proliferation and differentiation of neuronal cells.	[78]
MCPH11	*PHC1*	12p13.31	Nucleus	Regulates cell cycle, *PHC1* mutation highlights the role of chromatin remodelling in the pathogenesis of Primary Microcephaly.	[80]
MCPH12	*CDK6*	7q21.11	Cytoplasmic/nuclear	Controls cell cycle/organises microtubules, *CDK6* mutation affects apical neuronal precursor cells proliferation/reduces progenitor pool/decreases neuronal production/primary microcephaly.	[83]

Abbreviations. MCPH: Autosomal recessive primary microcephaly; MCPH1: Microcephalin; *WDR62:* WD repeat-containing protein 62; *CDK5RAP2: C*yclin dependent kinase-5 regulatory subunit associated protein*; CASC5:* Cancer susceptibility candidate-5; *ASPM:* Abnormal spindle like primary microcephaly; *CENPJ*: Centromere-associated protein J; *STIL:SCL/TAL1* interrupting locus; *CEP135:* Centrosomal protein 135; *CEP152:* Centrosomal protein 152*; ZNF335*: Zinc Finger Protein 335; *PHC1*: Polyhomeotic-like protein 1; *CDK6*: Cyclin-dependent kinase 6.

**Table 2:** Number of reported mutations in MCPH genes in families of different ethnic backgrounds worldwide.

MCPH genes	Reported mutations	Types of mutations				Ethnicity (families)	References
		Missense/nonsense	Splicing	Deletions & insertions	Complex rearrangements		
MCPH1	24	8	1	14	1	Iranian (8), Caucasian (1), Pakistani (13)	[5,22,51,86,89-91]
*WDR62*	28	14	1	13	0	Iranian (3), Turkish (11), Mexican (1), Arab (1), Pakistan (9)	[7,13,30,86,91]
*CDKRAP2*	6	3	2	1	0	Pakistani (2), Somalian (1)	[7,32,34,36,92]
*CASC5*	1	1	0	0	0	Moroccan (3)	[43]
*ASPM*	104	48	7	48	1	Iranian (13), Arab (12), Indian (8), European (22), African (5), Pakistani (60), many sporadic cases.	[7,14,50,51,86,91,93]
*CENPJ*	5	2	1	2	0	Iranian (5), Pakistani (6)	[32,60,61,86,91]
*STIL*	4	2	1	1	0	Iranian (2), Indian (3)	[68,86,87]
*CEP135*	1	0	0	1	0	Pakistani (1)	[20]
*CEP152*	9	5	2	2	0	Pakistani (5), Canadian (3)	[77,91,94]
*ZNF335*	2	1	1	0	0	Arab Israeli (1)	[78]
*PHC1*	1	1	0	0	0	Saudi Arabian (1)	[80]
*CDK6*	1	1	0	0	0	Pakistani (1)	[83]

Abbreviations. MCPH: Autosomal recessive primary microcephaly; MCPH1: Microcephalin; *WDR62:* WD repeat-containing protein 62; *CDK5RAP2:* Cyclin dependent kinase-5 regulatory subunit associated protein*; CASC5:* Cancer susceptibility candidate-5; *ASPM:* Abnormal spindle like primary microcephaly; *CENPJ*: Centromere-associated protein J; *STIL:SCL/TAL1* interrupting locus; *CEP135:*Centrosomal protein 135; *CEP152:* Centrosomal protein 152*; ZNF335*: Zinc Finger Protein 335; *PHC1*: Polyhomeotic-like protein 1; *CDK6*: Cyclin-dependent kinase 6.

**Table 3:** MCPH animal models.

MCPH gene	Animal orthologs of human gene	Animal model	References
*MCPH1*	*mcph1-/-* (Drosophila/mice)	Premature condensation of chromosome, non-coordinated centrosome, genome instability, detached centrosome, changes in cell cycle progression and defects in DNA damage repair, misregulated mitotic chromosome condensation.	[25,26,29]
*CDK5RAP2*	*Cnn-/-* (Drosophila)	Centrosome dysfunction causes connection loss between centrosomes and pericentriolar matrix. Only subtle defects of asymmetric divisions; the sizes of the observed brains were normal.	[39]
*ASPM*	*asp-/-* (Drosophila) *Aspm*-/-(mice)	The Drosophila *asp* gene is vital for both the organisation and binding together of microtubules at the spindle poles and to focus the poles of the mitotic spindles during mitosis and meiosis. Two mutant mouse lines showed that mutations in *ASPM* decreased the brain size in mice.	[56-58]
*CENPJ*	*Sas-4-/-*(Drosophila, *c. elegans*)	Centrioles are lost due to damage of the *CENPJ* orthologue *dsas*-4 in Drosophila; in contrast, the knockout flies can live until adulthood but have weak coordination and lower viability. The morphological development of mutant flies is normal without cilia or flagella, but they die in early life.	[65,66]
*STIL*	*STIL Sil-/-*(mice)	*STIL* knockout mice (*Sil-/-*) exhibit numerous developmental abnormalities in embryonic days E7.5-8.5 and die after E10.5, the phenotype of the mutant mice is characterised by decreased brain size, restricted development, defective midline neural tube, abnormal development, and enhanced apoptosis; the observed mutants were embryonically lethal.	[71]
*CEP135*	*Bld10-/-*(*Chlamydomonas*)	In Chlamydomonas, *CEP135* ortholog *Bld10p* mutants (*bld10*) demonstrated abnormal interphase microtubules and mitotic spindles, defects during cell division and a significant decrease in the growth rate.	[73]
*CEP152*	Orthologous (Drosophila asterless; *asl*)	*CEP152* is the putative mammalian orthologue of *Drosophila asterless*, mutations in which affect mitosis in the fly.	[77]
*ZNF335*	Orthologue (mice; *ZNF335*)	Knockdown of *ZNF335* caused a small brain size with an absent cortex and disrupted the proliferation and differentiation of neuronal cells. It was observed that knockdown of the *ZNF335* ortholog in mice (*ZFP335*) resulted in early embryonic lethality at day E7.5. Conditional knockdown of *ZNF335* in mouse cortical cells resulted in a small brain with a fundamentally absent cortex with deficient cortical neurons.	[78]

Abbreviations. MCPH1: Microcephalin; *CDK5RAP2:* Cyclin dependent kinase-5 regulatory subunit associated protein*; ASPM:* Abnormal spindle like primary microcephaly; *CENPJ*: Centromere-associated protein J; *STIL: SCL/TAL1* interrupting locus; *CEP135:* Centrosomal protein 135; *CEP152:* Centrosomal protein 152*; ZNF335*: Zinc Finger Protein 335.

### 1- Microcephalin (MCPH1)

MCPH1 encodes the BRCT (BRCA1 C-terminus) domain-containing protein microcephalin; microcephalin contains three BRCT domains, which are evolutionarily conserved phospho-peptide-interacting amino acid tandem repeat domains and are significant in cell cycle control. MCPH1 is characterised by a congenital decrease in brain size, especially in the cerebral cortex. The microcephalin gene is localised on chromosome 8p23, is composed of 14 exons and 835 amino acids, and has a genome size of 241,905 bp, with three already known isoforms and an 8,032 bp open reading frame [17,21]. The first documented MCPH1 gene mutation was observed in Pakistani families [21]; additionally, a large deletion mutation covering the first 6 exons of the microcephalin gene was reported in an Iranian family that exhibited autosomal recessive mental retardation and mild microcephaly [22]. An observed missense mutation in MCPH1 demonstrated a less severe cellular phenotype and mild microcephaly [5,7]. Later, a mutation in the same gene was identified in patients with microcephaly disorder, a misregulated chromosomal condensation termed premature chromosome condensation (PCC) syndrome and short stature [23,24]. A higher expression of this gene was observed in the fetal mouse brain during neurogenesis, especially in ganglionic eminences and lateral ventricles [21]. It was revealed that both MCPH1 and the PCC syndrome are allelic disorders originating from mutations within the same gene [4]. This protein is also involved in DNA damage-induced cellular responses, chromosomal condensation and during G2/M checkpoint arrest through maintaining the inhibitory cyclin-dependent kinase phosphorylation [6]. Further studies revealed that the consequences of MCPH1 deficiency in Drosophila include a slow mitotic entry, PCC, mitotic entrance with non-replicated DNA, non-coordinated centrosome, genome instability and a detached centrosome. Additionally, mitotic arrest was observed in Drosophila cells with deficient MCPH1 [25,26]. It was also observed that because of the influence on premature neurogenic formation, the progenitors with reduced MCPH1 increased the production of early born neurons, which comprise the deep layers (IV–VI), and decreased the late-born neurons, which produce the thinner outer cortex layer (II–III). However, neuronal migration is not affected due to MCPH1 deletion [27]. The successful generation of a mouse model for MCPH1-related primary microcephaly revealed misregulated mitotic chromosome condensation due to defective MCPH1 function and a reduced skull size [28,29].

### 2- *WDR62* (MCPH2)

The *WDR62* gene is localised inside the MCPH2 locus at chromosome 19q13.12 and has 32 exons and a genomic size of 50,230 bp [30]. *WDR62* is a WD40 repeat-containing protein expressed in neuronal precursors and post-mitotic neurons in the developing brain and is localised in the spindle poles of dividing cells. Phenotypic variations of *WDR62* advocate its key role in numerous aspects of cerebral cortical development [13]; it has been linked with severe brain malformations. A previous study revealed five homozygous *WDR62* mutations, including deletions and premature terminations, in patients of Turkish origin suffering from severe brain malformations and microcephaly [30]. A 7.5 Mb locus on chromosome 19q13.12 composed of 148 genes was recognised in two families via genome-wide linkage analysis. Additionally, high throughput sequencing of associated genes in each family produced more than 4,000 DNA variants harbouring deleterious changes involving a single gene *WDR62*. Additionally, six mutations were observed in the *WDR62* gene through targeted high throughput sequence analysis in six families suffering from a congenital microcephaly syndrome and diverse defects in cerebral cortical architecture [13]. Published observations from multiple groups have shown that *WDR62* is mainly nuclear in its location and lacks any apparent association with the centrosomes. In contrast to this, other findings have shown that the *WDR62* expression is similar to *ASPM*, which is also a MCPH protein focused at the spindle poles in neuronal precursor cells [30]. Another study further confirmed that disturbance of the centrosome integrity and spindle defects are the key players in developing MCPH2 microcephaly [31]. In HeLa cells, *WDR62* accumulates in the centrosome or nucleus in a cell cycle-dependent manner; siRNA knockdown of *WDR62* in cortical progenitors decreased their proliferation, caused defective spindle orientation, and reduced the centrosome integrity, displacement from the spindle poles and late mitotic development [31].

### 3- *CDK5RAP2* (MCPH3)

Homozygous mutations of cyclin-dependent kinase 5 regulatory subunit-associated protein-2 (*CDK5RAP2*) have been recognised as the basis of MCPH3 [32]. This protein is composed of 38 exons encoding 1,893 amino acids. Three *CDK5RAP2* mutations (two in Pakistani families; one in a Somalian patient) have been described; 1: a nonsense mutation in exon 4 (246T>A); 2: the A to G transition in intron 26 (4005-15A>G) produces a novel splice acceptor site, frame shift and a premature stop codon; and 3: a nonsense mutation in exon 8 (700G>T). All of these mutations result in a truncated protein with *CDK5RAP2* functional loss [32-34]. *CDK5RAP2* is strongly linked with the centrosome, Golgi apparatus, and microtubules and is chiefly present in the neural progenitors of the ventricular and sub-ventricular zones of an immature brain; it has also been observed in glial cells and early born neurons and is progressively down regulated as the brain matures [35,36]. *CDK5RAP2* is also essential in the regulation of the spindle checkpoint; its loss causes chromosomal missegregation and decreased spindle checkpoint protein expression through binding with their promoters and transcriptional regulation in HeLa cells [37]. A model of the microcephaly phenotype due to a mutation in *CDK5RAP2* causes a premature transition from symmetric to asymmetric neuronal progenitor cell division with a consequent reduction of the progenitor pool, decreased neurons and reduced cell survival [35,38]. Centrosome dysfunction, which is known as centrosome ‘rocketing’, was observed within the cells because of the connection loss between the centrosomes and pericentriolar matrix in the human *CDK5RAP2* ortholog ‘centrosomin’-deficient Drosophila embryos (cnn-/-). Only subtle asymmetric division defects were observed in ‘centrosomin’-deficient Drosophila embryos (cnn-/-), and the brain sizes were normal [39].

### 4- *CASC5* (MCPH4)

*CASC5* is a 265 KDa large protein that belongs to the conserved KMN protein network and permits chromosomal kinetochore docking to the microtubule. Its location adjacent to the kinetochore remains constant from G2 until late anaphase; proper docking is very important for sufficient sister chromatid segregation at anaphase and is vital for the spindle checkpoint of the mitotic cycle [40,41]. A *CASC5* mutation on the MCPH4 locus with an LOD score of > 6 in MCPH patients from three consanguineous families has been reported. This mutation enhanced the skipping of exon 18, produced a frame shift and premature stop codon in exon 19, followed by truncation of the predicted protein. Mutation 6125G>A in *CASC5* covers the amino acids from 1,981 to 2,108, which interact with ZWINT-1, whereas the *CASC5* C-terminus is important for binding with NSL1 and the MIS12 complex in a downstream portion of the mutation. Therefore, the loss of exon 18 and the resulting frame shift and truncation lead to a functional loss of *CASC5*[40,42]. This protein is also upregulated in the ventricular zone of the human fetal brain and interacts with BUBR1, ZWINT-1 and MIS12 through the N-terminal domain, which can be truncated due to mutation [43]. A transcriptome analysis through RNA sequencing of the germinal zone of the human fetal neocortex at 13-16 gestational weeks revealed a higher expression of *CASC5* in the ventricle zone compared the sub ventricle zone and cortical plate; this finding further confirmed the effect of mutated *CASC5* on proliferating cells in the ventricle zone that cause microcephaly [43,44]. Further analysis by *CASC5* siRNA knockdown in HeLa cells revealed chromosomal misalignment and the premature entry of the cells into mitosis; this defect causes improper symmetrical and asymmetrical division, which results in inefficient proliferation, inappropriate neuronal number and abnormal brain size [45].

### 5- *ASPM* (MCPH5)

Abnormal spindle-like primary microcephaly (*ASPM*), which is homologous to Drosophila melanogaster, is important for the normal functioning of the mitotic spindle in embryonic neuroblasts harbouring MCPH5 locus [14]. It consists of 62,567 bp with a 10,906 bp ORF, 28 exons and 3,477 amino acids, with a putative microtubule-interacting domain at the N-terminus [46,47], calponin homology domain, 81 isoleucine glutamine motifs acting as a calmodulin binding domain [14,48,49], and a C-terminus without any identified domains [50]. Studies have revealed four novel mutations of this gene in 33 families from Pakistan [51]. *ASPM* gene mutations truncate the protein and include deletion, single base pair change, duplication and variation in the intronic region. Additionally, substantial numbers of the observed *ASPM* mutations are compound heterozygous [10,52]. In neurogenesis, this gene is mainly expressed in the cerebral cortical ventricular zone and proliferation zones of the medial and lateral ganglion [14]. Another study showed the localisation of this protein at the spindles, and its knockdown by siRNA revealed reduced proliferative symmetric division in the mouse developing neocortex [53]. Studies have shown that because of neurosphere differentiation, *ASPM* is downregulated in mice; additionally, neurosphere proliferation and self-renewal was decreased as a result of *ASPM* knockdown [54]. Morpholino-mediated knockdown of *ASPM* in zebrafish resulted in a significant reduction in head size [55]. Drosophila *asp* gene mutants were the first to be known as having a mini-brain; this *asp* gene is known to be vital for both the organisation and binding of microtubules at the spindle poles and its ability to focus the poles of mitotic spindles during mitosis and meiosis [56,57]. Additionally, two mutant mouse lines were generated from gene trap cells for the study of *ASPM* function in the development of the cerebral cortex. The results showed that mutations in *ASPM* decreased the size of brain in mice [58]. It is therefore concluded that *ASPM* mutations can decrease the size of the brain by influencing the orientation of the mitotic spindle and can decrease the neuronal cells by affecting the asymmetrical to symmetrical cell division ratios [54,57].

### 6- *CENPJ* (MCPH6)

Mutations in the centromere-associated protein J (*CENPJ*) gene harboured in the MCPH6 locus on human chromosome 13q12.2 have been reported to cause primary microcephaly [32]. This gene is composed of 40,672 bp with a 5,187 bp ORF [46] and 17 exons, which encode a protein of 1,338 amino acids; it contains 5 coiled-coiled domains, many protein phosphorylation sites, 21 nonamer G-box repeats in the C-terminal domain and, C-terminal to that, contains a leucine zipper motif [32,59]. In total, four mutations were identified in the *CENPJ* gene: one mutation in one Brazilian family, [32] and one mutation in two Pakistani families [32]. Later, another Pakistani family showed the deletion of four consecutive nucleotides (TCAG) in the 11^th^ exon of the *CENPJ* gene, which causes a frame shift and a premature termination codon 19 bp downstream in the same exon that is predicted to add six amino acids downstream of the mutation [60]. The fourth mutation is a splicing mutation in the *CENPJ* gene that was identified in Seckel syndrome patients [61]. This protein is mainly expressed in the neuroepithelium of the frontal cortex at the beginning of neurogenesis [32]. Depletion of this protein disturbs the centrosome integrity, and cells deficient in *CENPJ* become arrested during mitosis, with multipolar spindles [62]. An *in vitro* analysis showed that this protein can impede microtubule nucleation and depolymerisation, predicting a role for *CENPJ* in directing the centrosomal assembly of microtubules [63]. Prior studies have also shown that *CENPJ* deletion by siRNA causes the enhancement of multiple spindle poles, apoptosis and mitosis arrest [64]. Centrioles are lost due to damage of the *CENPJ* orthologue *dsas*-4 in Drosophila; in contrast, the knockout flies can live until adulthood but have weak coordination and lower viability [65]. The morphological development of mutant flies is normal without cilia or flagella, but they die in early life [66]. *Sas*-4 is a centriole protein controlling centrosome organisation in *C. elegans*, and its involvement has been confirmed in the duplication of centrosome fluorescence recovery after photo bleaching (FRAP). *CENPJ* regulates the centriole length throughout biogenesis, with possible involvement in the accurate recruitment of centriolar microtubules [67].

### 7- *STIL* (MCPH7)

The *SCL/TAL1* interrupting locus (*STIL*) gene was identified at the seventh locus for MCPH, spanning 63,018 bp with a 5,225 bp ORF, 20 exons and 1,287 residues in a full-length cytosolic protein of 150 kDa; it also contains a putative nuclear localisation signal and C-terminal domain [17,68]. Three mutations causing protein truncation were observed in members of five Indian families in an 8.39 Mb region of chromosome 1p33-p32.3 [68]. The expression of this gene is observed throughout the cytosol but mainly in the peri-nuclear region, with an essential role in mitotic entry (G2-M phase), apoptosis regulation and centrosome function [68]. *STIL* is a primary response gene that is ubiquitously expressed in proliferating cells and in early embryonic development [69,70]. Studies of zebrafish have revealed that *STIL* has an important role in the duplication of centrosomes and in the organisation of mitotic spindles. *STIL* mutation in zebrafish causes an embryonic lethal defect [69], and *STIL* knockout mice (*Sil-/-*) exhibit numerous developmental abnormalities at embryonic day E7.5-8.5 and die after E10.5. The phenotype of the mutant mice is described as a decreased size, restricted development, defective midline neural tube, abnormal development, enhanced apoptosis, reduced proliferation and abnormal expression of numerous essential genes, including lefty-2 and nodal. Additionally, the observed mutants were embryonically lethal [71].

### 8- *CEP135* (MCPH8)

Centrosomal protein (*CEP135*) is encoded by the *CEP135* gene, is composed of 26 exons and 1,140 amino acids and is a conserved α helical protein observed throughout the cell cycle at the centrosome. As a centrosomal component, this protein gives more strength to the centrosomes. A truncated mutation in *CEP135* was identified in a Pakistani family at locus 4q12 that caused microcephaly. A deletion mutation 970delC changes glutamine to serine at position 324, followed by the premature termination of a codon. Considering the mutation’s position and the affected protein; the truncated mutation is definitely not compatible with the normal function of *CEP135*[20]. Studies by electron microscopy revealed that *CEP135* is associated with pericentriolar material, an electron-dense material around the centrioles. In interphase, the mitotic spindles are disorganised due to an RNAi-mediated decreased amount of *CEP135* in the cells, which suggests that *CEP135* maintains the organisation and structure of the centrosomes and microtubules [72]. In Chlamydomonas, *CEP135* ortholog *bld10p* mutants (*bld10*) demonstrated abnormal interphase microtubules and mitotic spindles, defects during cell division and a significant decrease in the growth rate [73]; additionally, *CEP135* knockdown in *CHO* cells showed a decreased growth rate [72]. The C-terminal domain of *CEP135* contains an interacting site for its two binding partners, the 50 kDa dynactin complex subunit (p50) and C-Nap1. Due to the truncation of *CEP135*, both p50 and C-Nap1 are released, promoting the decomposition of centrosomes and the premature splitting of the centrosomes, respectively [74,75].

### 9- *CEP152* (MCPH9)

*CEP152* is an important 152 kDa centrosome protein that organises the microtubules of animal cells and has an essential role in giving shape to the cell, polarity, motility and cell division [76]. The *CEP152* gene is localised to the MCPH4 locus at chromosome 15q21.1; recently, microcephaly due to a mutation in the *CEP152* gene was designated as MCPH9 [43,77]. Human *CEP152* is an ortholog of the Drosophila asterless (*asl*) gene, contains 72,835 bp and 1,710 amino acids encoding a 152 kDa protein [77]. Three families with one microcephalic child each were identified from a subpopulation of eastern Canadian. A homozygosity analysis of two of the families by genome-wide dense SNP genotyping revealed the locus 15q21.1 chromosome. Exon sequencing of the candidate genes identified a non-conserved amino acid conversion into a highly conserved *CEP152* residue [77]. One mutation converts glutamine into proline, which is anticipated to be pathogenic and can disturb a potential coiled-coiled protein domain. The other mutation causes protein truncation by eliminating a portion predicted to be a coding region from the C-terminal domain [77]. A greater head size reduction was observed in compound heterozygous females compared with missense homozygous females. The truncated protein causes a nonsense-mediated decay of the mutated transcript; studies have also revealed during a functional assay to observe the subcellular localisation that the wild type *CEP152*-GFP fusion protein can be identified in γ-tubulin-co structures [77]. Mutant *CEP152* flagged with GFP failed to colocalize with the γ-tubulin, which further confirmed the pathogenicity due to this mutation [77].

### 10- *ZNF335* (MCPH10)

The *ZNF*335 gene encodes a component of the vertebrate-specific, trithorax H3K4-methylation chromatin remodelling complex, which regulates neuronal gene expression and cell fate [78]. MCPH10 was identified due to a mutation in the *ZNF335* gene in affected members of an Arab Israeli family. This mutation was recognised in a homozygous 3332G-A transition in exon 20 of the *ZNF335* gene and resulted in an Arg1111 to His (R1111H) substitution at a conserved residue in the 13th zinc-finger domain. This mutation was identified at the final position of a splice donor site that disturbed normal splicing, leading to an abnormally large transcript that included introns 19 and 20 with a predicted premature termination [78]. Patient cells also showed severely reduced levels of *ZNF335* protein, but a few residual transcripts were identified. Cellular studies revealed that *ZNF335* binds to the chromatin remodelling complex, including H3K4 methyl transferases that can regulate the expression of specific genes in various pathways; the complex is analogous to the TrxG (trithorax) complex in Drosophila. Mutations in nBAF, one of the neural-specific chromatin regulatory complexes, affected proliferation, revealing that it is related to microcephaly [79]. Insufficiency of the *ZNF335* gene causes the degeneration of neurons, which makes it different and much more severe compared with other microcephaly syndromes that are well linked to postnatal survival. *In vitro* and *in vivo* mouse models showed that knockdown of *ZNF335* caused a small brain size with an absent cortex and disrupted the proliferation and differentiation of neuronal cells [78]. It was observed that knockdown of the *ZNF335* ortholog in mice (*ZFP335*) resulted in early embryonic lethality at day E7.5. Conditional knockdown of *ZNF335* in mouse cortical cells resulted in a small brain with a fundamentally absent cortex with deficient cortical neurons. Knockdown of *ZNF335* in mouse neuronal progenitor cells caused reduced proliferation and self-renewal due to premature cell cycle exit. Similar findings were observed in an *in vivo* embryonic mouse model using *in utero* electroporation to target *ZNF335* in cortical progenitor cells. *ZNF335* depletion resulted in abnormal neuronal cell orientation and radial glia with disorganised dendritic outgrowth. [78].

### 11- *PHC1* (MCPH11)

Primary microcephaly-11 (MCPH11) is caused by homozygous mutation in the *PHC1* gene on chromosome 12p13. A 12-year-old girl and her 6-year-old brother, who were born to related Saudi parents, suffered from primary microcephaly (-5.8 and -4.3 SD, respectively) and low normal cognitive function. The brain MRIs were normal, except for a small brain size. The sister and brother also had short stature (-3.6 SD and -2.3 SD, respectively) [80]. The mutation, which was found by homozygosity mapping combined with exome sequencing, segregated with the disorder and was not found in the dbSNP, Exome Variant Server or 1000 Genomes database or in 199 Saudi exomes or 554 Saudi control individuals [80]. A microarray analysis showed that various genes with a role in regulating the cell cycle are dysregulated in the patient cells [80]. Cells with a *PHC1* mutation also enhanced the geminin expression, which was recapitulated in RNAi experiments in control and patient cells in which *PHC1* was ectopically expressed. Because geminin has a recognised role in controlling the cell cycle [81,82], PM pathogenesis could be partly due to abnormalities in the cell cycle that are the result of accumulated geminin. The patient cells also showed an increase in DNA damage and defective DNA repair in response to irradiation and abnormal cell cycle activity consistent with reduced proliferative activity compared with controls. These defects were associated with abnormalities in chromatin regulation and could be rescued in patient cells by the overexpression of wild type *PHC1*[80].

### 12- *CDK6* (MCPH12)

The cyclin-dependent kinase 6 (*CDK6*) protein controls the cell cycle and differentiation of different cell types. Prior studies showed a genetic defect through a novel MCPH locus on the long arm of chromosome 7 (MCPH12). This mutated gene causes disorganisation of the microtubules and spindles, distorted nuclei, and supernumerary centrosomes. Furthermore, this role was confirmed by knockdown cells and cells expressing the *CDK6* mutant allele, whereas the decreased proliferation observed in the patient and knockdown cells reinforced its already described role in controlling the cell cycle [83]. *CDK6* mutation also affects apical neuronal precursor cell proliferation and produces an imbalance between symmetric and asymmetric cell division, which results in a progenitor pool reduction that could be the reason of decreased neuronal production and, finally primary microcephaly [83]. A study of a consanguineous eight-generation Pakistani family with ten microcephalic children showed a new MCPH locus at HSA 7q21.11-q21.3 [83]. Most related candidate genes in this region showed a homozygous single nucleotide substitution, 589G>A, in *CDK6*, which encodes cyclin-dependent kinase 6. This mutation leads to an abnormal mitotic morphology and has an effect on cellular localisation because patient primary fibroblasts failed in recruiting *CDK6* to the centrosome during the mitosis process [84]. This finding was confirmed by another study that showed a lower proliferation capacity of CDK6 patient primary fibroblasts and knockdown cells; CDK6 also has a significant role in differentiating various cell types [85].

## Mutation spectrum of MCPH Genes in Middle Eastern and Saudi populations

A total of approximately forty-three families have been reported with mutations in MCPH gene from the Middle Eastern and, especially, Saudi Arabian region: twenty-eight from Iran, fourteen from Arab countries, three from Morocco and one from Saudi Arabia [22,43,80,86,87] . More specifically, eight Iranian families have been reported with mutations in the MCPH1 gene, thirteen with mutations in the *ASPM* gene, five with mutations in the *CENJP* gene and two with mutations in the *STIL* gene [22,86,87]. CASC5 gene mutation have been found in MCPH patients from three consanguineous Moroccan families with a homozygous missense mutation 6125G>A that leads to an amino acid substitution Met2041Ile; this substitution is predicted to inactivate an exonic splicing enhancer (ESE), leading to an abnormal transcript lacking exon 18 [43]. A total of thirteen Arab families have been reported with mutations in the *ASPM* and *ZNF335* genes. A homozygous 2974C>T mutation in the *PHC1* gene on chromosome 12p13 was reported in a 12-year-old girl and her 6-year-old brother born to related Saudi parents who were suffering from primary microcephaly [80].

## Conclusions

Cognitive capabilities are reduced with a substantial decrease in brain size in MCPH patients, whose brain sizes decrease to one third compared with its original size with a substantial cognitive decline. There are twelve known genes that cause this neurodevelopmental disorder, and recent evidence suggests that MCPH is possibly a primary disorder of neurogenic mitosis. With the progress of MCPH gene identification, prenatal diagnosis (detects a disorder recurrence), postnatal diagnosis (differentiates the disorder from various differential diagnosis) and carrier testing (for consanguineous families in which the disease is known to occur) are increasingly available for patients. The successful creation of mammalian models for MCPH has enabled researchers to further add to the knowledge regarding the aetiology and pathophysiology of MCPH. Better genotyping, neuro-physiological and neuroimaging testing, together with the creation of genetically homogeneous groups of patients, would benefit the identification of exact genotype phenotype correlations. Mutational screening in different MCPH families from the Middle East and especially Saudi Arabia would help in genetic counselling and prenatal diagnosis for MCPH and would eventually enable us to reduce the incidence of MCPH in a highly consanguineous population. Additionally, these studies have increased our understanding of the control of neuronal production by neural stem cells and how this has been modulated by evolution to control the brain sizes of different species.

## Abbreviations

MCPH: Autosomal recessive primary microcephaly; OFC: Occipitofrontal circumference; SD: Standard deviations; HC: Head circumference; PCC: Premature chromosome condensation; MRI: Magnetic Resonance Imaging; *WDR62:* WD repeat-containing protein 62; *CDK5RAP2*: Cyclin dependent kinase-5 regulatory subunit associated protein-2; *CASC5:* Cancer susceptibility candidate-5; *ASPM:* Abnormal spindle like primary microcephaly; *CENPJ:* Centromere-associated protein J; FRAP: Fluorescence recovery after photo bleaching; *CEP:* Centrosomal protein; *ZNF335*: Zinc Finger Protein 335; *PHC1*: Polyhomeotic-like protein 1; *CDK6:* Cyclin-dependent kinase 6.

## Competing interests

The authors declare that they have no conflicts of interest.

## Authors’ contributions

MF, MIN and PNP wrote the manuscript. MR, AGC, TAK, MHA, AMI, HSJ and FA edited the final version. All authors read and approved the final version of the manuscript.

## Declarations

This article has been published as part of *BMC Medical Genomics* Volume 8 Supplement 1, 2015: Selected articles from the 2nd International Genomic Medical Conference (IGMC 2013): Medical Genomics. The full contents of the supplement are available online at http://www.biomedcentral.com/bmcmedgenomics/supplements/8/S1
